# Influence of Driving Pulse Properties on Third-Harmonic Diffraction from Quasi-BIC Metasurfaces

**DOI:** 10.1021/acsphotonics.5c01526

**Published:** 2025-11-12

**Authors:** Falco Bijloo, Arie J. den Boef, Peter M. Kraus, A. Femius Koenderink

**Affiliations:** † 55952 Advanced Research Center for Nanolithography, Science Park 106, 1098 XG Amsterdam, The Netherlands; ‡ Department of Physics of Information in Matter and Center for Nanophotonics, NWO-I Institute AMOLF, Science Park 104, 1098 XG Amsterdam, The Netherlands; ¶ Department of Physics and Astronomy, and LaserLaB, Vrije Universiteit, 1081 HV Amsterdam, The Netherlands; § ASML Netherlands B.V., 5504 DR Veldhoven, The Netherlands

**Keywords:** quasi-BIC, nonlinear metasurface, tunable diffraction, Fano resonance, driving pulse, third-harmonic generation

## Abstract

Quasi-bound states in the continuum in dielectric metasurfaces support sharp Fano resonances that emerge from the interference between bright and dark modes. We exploit this modal interplay to demonstrate tunable third-harmonic emission, controlled through the driving pulse’s wavelength and intensity. Our experiments show imbalances in third-harmonic diffraction patterns and non-Gaussian third-harmonic spectral features that exhibit strong variations near the Fano resonance. We explain the observations via a coupled-oscillator model that captures the interplay between the driving field and the nonlinear response of the modes, explaining our observations and providing a predictive framework for optimizing the third-harmonic diffraction efficiency. These results establish pulse-engineered metasurfaces as a powerful platform for nonlinear wavefront shaping and frequency conversion applications while simultaneously serving as a warning that pulse properties play a vital role in metasurface function design.

## Graphical abstract

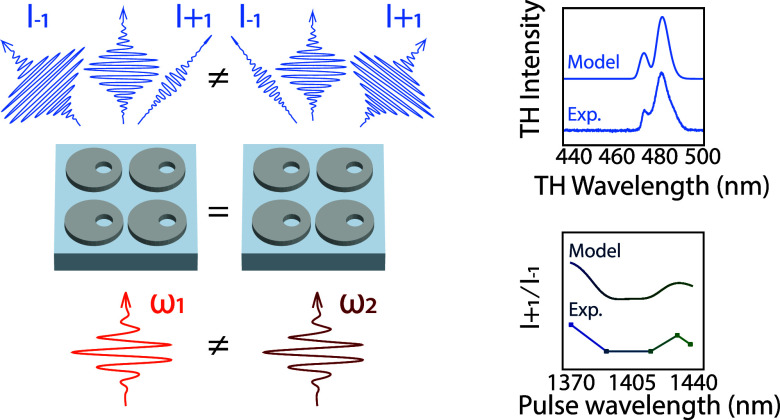

## Introduction

Over the past decade, nonlinear optical metasurfaces have emerged as an effective means to study fundamental light–matter interactions^1^ and they are pursued for a range of applications,^2^ such as efficient generation of structured beams of harmonic light,^3–5^ tunable functionality,^6–8^ and analog signal processing with nonlinear films.^9^ The ability to design nanoscale meta-atoms with tailored resonance wavelengths, quality factors, and polarization properties has stimulated significant progress in optimizing nonlinear functionality. Resonant effects, in particular, are known to enhance conversion efficiencies by amplifying local near-fields.^10^ Among these, quasi-bound states in the continuum (quasi-BIC) resonances have demonstrated exceptional performance for nonlinear frequency conversion, employing Fano lineshapes that significantly boost third-harmonic and high-harmonic generation (THG and HHG) by many orders of magnitude.^11–13^ These quasi-BIC resonances can occur when a dark mode (bound state) is coupled to a bright mode (acting as a continuum contribution) to give a Fano resonance in linear response.^14^ Nonlinear metasurfaces not only boost conversion efficiencies in solid-state systems by 3–5 orders of magnitude compared to those in unpatterned films; one can also imprint nontrivial wavefronts, encoded in the arrangement and shape of the meta-atoms. This can find applications in areas such as nonlinear holograms^15^ and beam shaping^5^ to obtain beams with orbital angular momentum and nontrivial vector properties, beam steering,^6,16–18^ imaging,^19^ and dynamic image tuning,^4^ demonstrating their versatility in both fundamental science and technological applications.

Most of the work in this domain has focused on enhancing nonlinear conversion efficiencies and shaping the desired beam properties^20^ by leveraging meta-atom design, while usually not considering in detail the role of the input pulse shape. It is well understood that conversion efficiencies are highest when input pulse bandwidths match the quality factors of quasi-BIC resonances, while not much research has been attributed to understand how to shape nonlinear output as a function of other driving pulse properties. For high driving powers, it is reported by multiple groups that metasurfaces for nonlinear conversion suffer from saturation effects.^12,18,21^ Such saturation effects can occur due to self-action (e.g., refractive index tuning and absorption induced by frequency-converted light) and can lead to limited conversion efficiencies, as well as power dependence of the generated TH and HH spectra. While it has been reported that such nonlinear effects could be useful for *manipulating* the harmonic generation process,^21^ in the majority of studies, the critical role that driving pulse parameters play in shaping nonlinear optical emission is either overlooked or seen as a limiting factor, and certainly not seen as a resource.

In this article, we report on an experimental study of nonlinear emission from Fano resonant metasurfaces as a function of driving pulse properties, and we show that nonlinear diffraction patterns and spectra crucially depend on multimode interference. For a given, fixed metasurface, this means that very different TH conversion spectra and diffraction patterns can be obtained simply by choosing different input pulses. Our experiments are supported by a time-domain coupled oscillator model to provide insight into the key ingredients required to understand the vital influence of pulse properties on the behavior of diffractive nonlinear metasurfaces. Our findings highlight a pathway to more precise control over nonlinear emission by adjusting the characteristics of the excitation pulse, rather than by modifying the metasurface structure itself. This approach not only holds promise for fundamental research, such as exploring spectral-temporal studies of nonlinear diffraction, but also expands the potential for practical applications in tunable photonic devices, enabling nontrivial control over nonlinear emission profiles.^22,23^

## Results and Discussion

Figure 1 shows the main concept of our experiment. We excite a Fano resonant nonlinear metasurface with infrared pulses and study the generated TH diffraction patterns and spectra depending on how the excitation pulse matches the Fano resonance. To this end, we vary the metasurface geometry to control the Fano line width and resonant wavelength, we vary the laser tuning (within the 1320–1480 nm range) to control the detuning between excitation and metasurface resonance, and we perform measurements as a function of pulse power.

**Figure 1:**
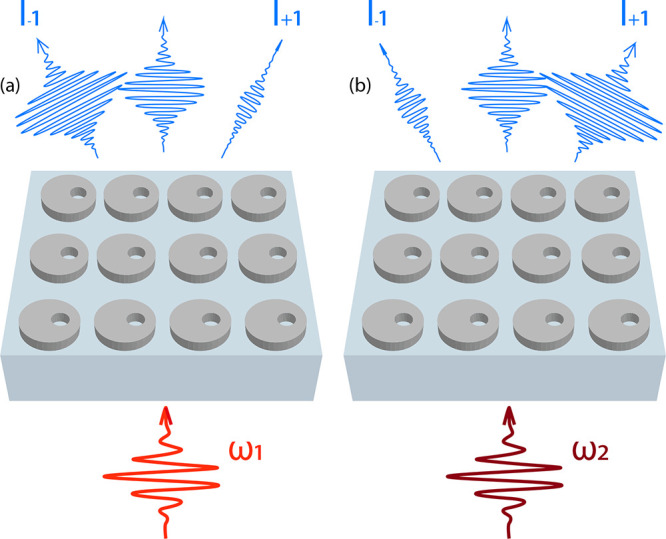
Schematic of our approach to studying the nonlinear response as a function of driving pulse power and frequency. An infrared driving pulse excites a Fano resonance supported by a quasi-BIC dielectric metasurface. Depending on the pulse properties, such as power and frequency, a strong contrast emerges between opposing TH diffraction orders. This effect is illustrated in panels (a) and (b), where different driving frequencies result in varying diffraction order intensities.

The dielectric metasurface (scanning electron micrograph shown in Figure 2a) is fabricated in polycrystalline silicon (75 nm thick) evaporated on fused quartz, patterned with electron beam lithography and subsequent reactive ion etching. The Supporting Information (SI) reports in detail on the nanofabrication procedure. The sample consists of a square grid (880 nm pitch) of nominally identical unit cells that consist of a silicon disk with a radius of *r*_d_= 295 nm and an off-centered hole with a radius of *r*_h_ = 125 nm. When the hole is placed at the meta-atom center, the system supports a symmetry-protected BIC mode, which is unaddressable under normal incidence. This resonance is opened to coupling to far-field radiation by introducing asymmetry through off-center placement of the hole.^4,24,25^ The dimensionless asymmetry parameter α is defined as α = *d*/*r*_d_, where *d* is the distance between the hole and the center of the disk. The quasi-BIC mode manifests as a strong Fano resonance in transmission, resulting from the coupling between a bright in-plane electric dipole (ED) and a dark out-of-plane magnetic dipole (MD).^26^ Figure 2b presents a measured transmittance spectrum for a metasurface with α = 0.3 (green curve). The typical Fano line shape at ca. 1415 nm wavelength has a quality factor of ca. *Q* = 289, as extracted from fitting a coupled oscillator model (dark green dashed curve; see the SI for a detailed description of the coupled oscillator model). Figure 2c,d shows simulations (c) and experimental results (d) of the spectral response as a function of α, which increases from bottom to top on the vertical axis. The simulation evidences a strong redshift with increasing asymmetry, as well as a strong dependence of the quality factor of the quasi-BIC, which decreases with increasing asymmetry as expected. Experimental transmittance spectra as a function of asymmetry agree closely with the simulation, aside from a minor overall shift in the wavelength axis, which we ascribe to a refractive index mismatch between the simulated silicon (*n* = 3.45) and the evaporated poly-Si, and minor differences in geometry dimensions, such as hole radius.

**Figure 2:**
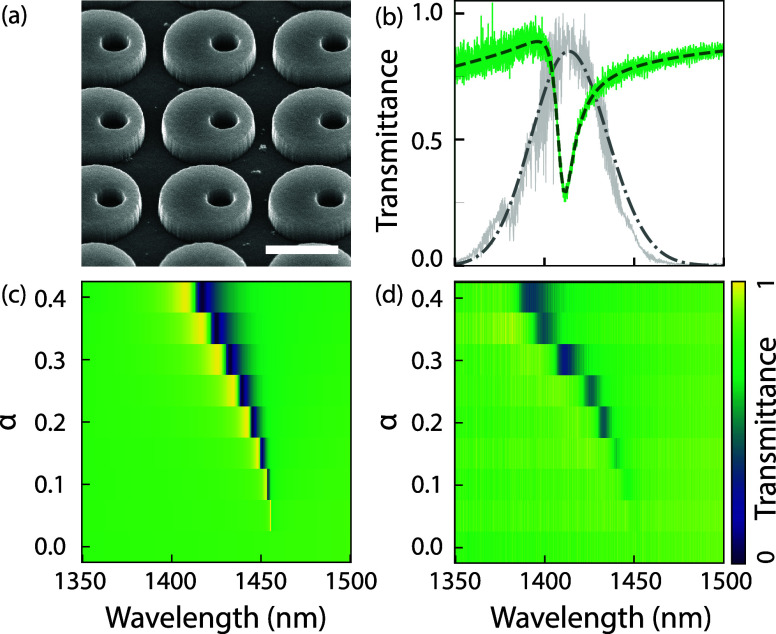
Metasurface properties: (a) Scanning electron micrograph of the silicon disk metasurface featuring an asymmetrically placed hole (scale bar 500 nm), pitch *p* = 880 nm, disk radius *r*_d_ = 295 nm, hole radius *r*_h_ = 50, and Si thickness *d* = 75 nm.) The center of the hole is positioned at a distance *l* = α*r*_d_ from the center of the disk, where the asymmetry parameter α ranges from 0 (center) to 1 (edge), with this metasurface having α = 0.3. (b) Linear transmittance of the metasurface (green solid line) with a coupled oscillator fit (dark green dashed), revealing a Fano line shape of quality factor *Q* = 289. For reference, a 130 fs chirped pulse (chirp rate β = −5 × 10^–6^ rad/fs^2^), centered at 1414 nm, used for third-harmonic generation (experimental gray solid, modeled Gaussian pulse with similar parameters in dark gray dashed-dotted line) is also shown. (c) COMSOL simulation of transmittance for a metasurface with similar properties, varying asymmetry parameters α = 0 to α = 0.4. The Fano resonance blueshifts and broadens, as the electric near-field concentrates in the hole for larger α; thus, the field experiences a lower refractive index and stronger coupling from the bound state to the continuum. (d) Experimental transmittance of the fabricated metasurfaces with α ranging from 0 to 0.4, demonstrating strong agreement with simulations.

The metasurfaces are studied in a femtosecond Fourier microscope (schematic setup in Figure 3a and more detailed setup in the SI). A train of 100 fs pulses at a 1 MHz repetition rate and a wavelength of 1030 nm from a LightConversion Pharos laser is fed (input power 8 W) into a LightConversion Orpheus-F hybrid optical parametric amplifier, which generates idler pulses of 130 fs in the wavelength region of interest for our experiments (1300–1500 nm), of which a typical pulse spectrum is shown in Figure 2b (shaded gray). A combination of a half-wave plate and a linear polarizer (Thorlabs AQWP10M-1600, LPVIS050-MP2) at the output of the laser controls the driving power, after which the pulse train is loosely focused onto the metasurface using an *f* = 30 mm lens, creating a spot with a radius of 11 μm on the sample. In the transmitted direction, we employ a 100× microscope objective (Nikon AC API plan, numerical aperture (NA) of 0.9) to collect transmitted infrared light and nonlinear emission. A dichroic mirror (Edmund Optics 69-900) reflects the third-harmonic light while transmitting the infrared beam for analysis on an optical spectrum analyzer to determine pulse central wavelength. To fully suppress the infrared beam in the third-harmonic detection path, we additionally use two short-pass filters (Thorlabs TF1). The third-harmonic emission is either fed into a grating spectrometer (Andor Shamrock 163i, 163 mm focal length, with a 300 lines/mm grating (SR1-GRT-0300–0500) and a 25 μm fixed-width vertical slit, onto which we mounted a monochrome CMOS camera (Ximea MC124MG-SY-UB) to resolve the third-harmonic spectrum or, alternatively, is directed onto a camera (Teledyne Prime BSI Express) to produce real- and Fourier-space images (selected by a flippable mirror, projecting the object plane or the back focal plane (BFP) on the camera chip). When pumped at full power (6 mW at 1 MHz repetition rate), bright TH is clearly visible, and a photograph of the TH on a piece of paper near the BFP of the objective (Figure 3a, right, taken with a phone camera) underlines both the strong THG conversion efficiency and the fact that the THG light is distributed over a set of diffraction orders. Here, it should be noted that the metasurface does not support diffraction orders for infrared excitation light. It is the induced polarization current at 3ω that can radiate into a set of diffraction orders, owing to the fact that the metasurface periodicity exceeds the third-harmonic wavelength. The typical conversion efficiency enhancements, compared to the conversion efficiency of unpatterned silicon film on the same sample, are η_enh_ = 10^3^ (measured at a pump power of 0.73 mW = 0.19 mJ/cm^2^ ≃ 1.37GW/cm^2^). Absolute conversion efficiencies (fundamental to harmonic power) are typically η ∼ 10^–6^ – 10^–5^.

**Figure 3:**
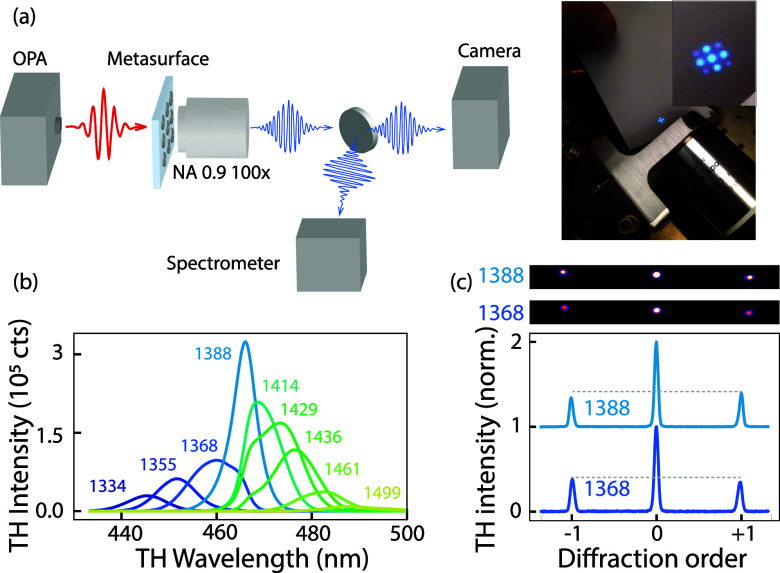
Third-harmonic generation experiment for studying TH spectra and TH diffraction contrast: (a) Sketch of the experimental setup to study TH spectra and diffraction contrast. An optical parametric amplifier (OPA) generates ca. 130 fs pulses centered between 1300 and 1500 nm, which are loosely focused (*f* = 30 mm) to excite the metasurface from the backside. A collection objective (NA 0.9, 100×) captures the TH light, projecting its back focal plane (BFP) via a 4*f*-telescope onto a camera. Alternatively, a flip mirror directs the TH beam into a spectrometer. The right image, captured by a phone camera just behind the BFP, shows a typical blue TH diffraction pattern. (b) TH spectra as a function of pulse tuning, with each color representing an excitation pulse centered at the wavelength indicated above the spectrum, for the metasurface with α = 0.3. The average power is maintained at ca. 0.7 mW, corresponding to a pump fluence of 0.15 mJ/cm^2^. (c) Summed counts of the −1, 0, and +1 TH diffraction orders for pulses centered at 1368 nm (bottom dark blue) and 1388 nm (top light blue), showing pulse tuning can inverse diffraction order intensities. Pulse input power is 3 mW (0.79 mJ/cm^2^).

Third-harmonic spectra for different tunings of the pump pulse, at constant pump power, are presented in Figure 3b (pulse power = 0.5 mW, corresponding to a pump fluence of 0.15 mJ/cm^2^), with different plot colors representing pulse central wavelengths, as indicated in the caption, for a metasurface with asymmetry parameter α = 0.3. Two key observations emerge. First, THG conversion is at a maximum when the pulse is tuned to spectrally match the Fano resonance. Second, the THG conversion spectra are not Gaussian, like the excitation pulse, but show marked shoulders. This is also observed in previous work.^4,21^ The spectrum is narrowest and most like a single peak when the excitation matches the Fano resonance. For detuning in either direction, the converted pulses are broader, appearing to show a component that is pinned to the Fano resonance and a component that tunes with the incident pulse. Figure 3c shows crosscuts of 2D camera images taken in Fourier imaging mode. The central peak represents the wavelength-integrated intensity of the zeroth diffraction order for the THG light, while the side peaks correspond to the ±first diffraction orders. These diffracted orders are diffracted along the direction of the structural asymmetry in the Fano resonant meta-atoms (also for a metasurface with α = 0.3), which is orthogonal to the polarization direction of the driving field (input power of 3 mW, which corresponds to ca. 0.79 mJ/cm^2^). The diffraction data have the following characteristics. First, the diffracted intensity is significant, with the intensity in the diffracted orders of up to 50% of the intensity in the zeroth order. Second, the intensity of the +first and −first diffraction orders is different, indicated by the gray horizontal dashed line. This is a direct consequence of the structural asymmetry, and we verified that the diffraction orders along the perpendicular axis do not show such an asymmetry. Finally, the bottom dark blue and top light blue lines represent crosscuts at different central wavelengths of the pump pulse (1368 and 1388 nm), clearly demonstrating that the asymmetry in the TH diffraction that arises from the structural asymmetry, in fact, depends on the pulse tuning. For this particular nanostructure, the TH contrast $\Delta_{I}=\frac{I_{+1}-I_{-1}}{I_{+1}+I_{-1}}$ in the asymmetry ranges from zero (no significant difference) to circa ±0.3, depending on pulse tuning and power.

A main conclusion drawn from the observations presented in Figure 3 is that nonlinear TH generation depends, in terms of efficiency, spectral content, and diffracted output pattern, on the tuning of the input pulse relative to the metasurface Fano resonance. Less obvious is if these phenomena show a trivial third-power dependence on pump fluence, or whether there, furthermore, is a nontrivial power dependence of THG spectra and diffraction patterns. Figure 4 presents a power-dependent study of TH spectra and TH diffraction contrast. In Figure 4a, we show TH spectra generated for two selected input pulse wavelengths 1388 nm (blue curve, at Fano resonance) and 1436 nm (green, red-shifted from Fano resonance) and at different input powers (lighter (darker) colors corresponding to lower (higher) input powers, ranging from 0.36 to 2 mW average power (corresponding to 0.09–0.53 mJ/cm^2^)). To focus on the spectral shapes, the spectra are normalized. Integrated intensity values are presented in panel (b). The spectra are clearly power-dependent. For the reddest pump wavelength, the shoulder at the Fano resonance observed in the tuning data (Figure 3b) is clearly present. Notably, its relative height compared to the main peak decreases with increasing input power (bright to dark green spectra in Figure 4a). For the bluer pump wavelength, it is obvious that the spectrum broadens. We interpret both sets as indicative of one and the same physical scenario: the emission consists of a superposition of two contributions. Of these, one is pinned to the Fano resonance frequency, which quickly saturates with increasing power, and it arises from the quasi-BIC contribution. The other contribution likely arises from the broad dipole mode of the meta-atom. Since the broad dipole mode hardly shows a frequency structure in its linear response, the third-harmonic spectrum that arises from its nonlinear response is essentially the third-harmonic spectrum of the pump pulse as it would appear in a nonresonant medium. We hypothesize that this contribution does not saturate as quickly with incident pump power as the quasi-BIC contribution. On the basis of this hypothesis, we fit the spectra to sums of two Gaussians, extracting the power-dependent growth of the integrated intensity of these two contributions. Example fits of the two fitted Gaussians are shown in the SI. The integrated TH intensity of the fitted Gaussians is plotted in Figure 4b, in blue (green) for the 1388 (1436) nm pulses, where the circular markers represent the main peak and the squares represent the shoulder at the Fano frequency. Interestingly, the off-resonance 1436 nm pulse demonstrates a clear third-order power law for the main peak (green circles) across most of the pump fluence range, while the shoulder (green squares) grows according to a second-order power law, above 0.3 mW. From around 2 mW onward, the dependencies deviate from both the third- and second-order power laws, both showing a saturation behavior. Both on-resonant 1388 nm pulse Gaussian intensities show a third-order law for the lower input powers, and saturation sets in at input powers of 1 mW, or even somewhat below. Saturation of spectrally integrated conversion efficiencies has been commonly reported in the literature of nonlinear metasurfaces and has been commonly attributed to self-action effects, such as free-carrier-induced detuning of the metasurface resonance.^4,21^ In Figure 3c, we plot TH diffraction contrast Δ_*I*_ as a function of pulse tuning for six different input powers (0.7–5.2 mW, in steps of 0.9 mW, corresponding to lighter to darker colors). The TH contrast is also plotted in gray for the vertical orders, hovering around 0 at all tunings and input powers, showing that diffraction asymmetry occurs only along the geometrical axis, along which the meta-atom symmetry is broken (slight deviations for the lowest input powers can be attributed to the larger noise level). The diffraction contrast is generally minimal when the pump pulse is tuned to the Fano resonance and reaches significant values upon detuning the pump from the Fano resonance. Moreover, the diffraction asymmetry generally depends on the pump power. In fact, at a given pump tuning, the diffraction asymmetry even *inverts* upon increasing the pump power.

**Figure 4:**
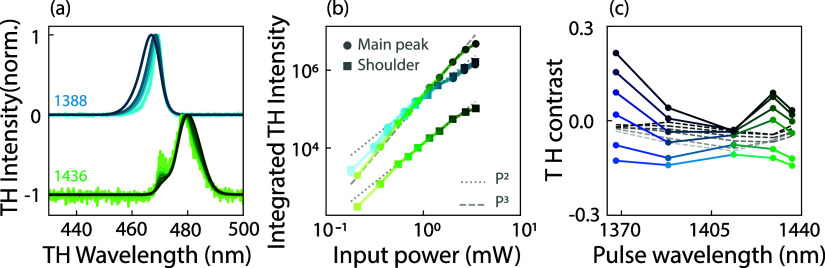
Power-dependent study of TH spectra and TH diffraction contrast: (a) Normalized TH spectra for pulses centered at 1388 nm (blue) and 1436 nm (green), with increasing pump power indicated by lighter to darker shades (0.36 0.55, 0.70, 0.88, 1.16, 1.47, and 2.01 mW). (b) Log–log plot of TH power as a function of input pulse power, displaying the integrated TH intensity for the two Gaussians for each pulse—one corresponding to the main peak and the other to the shoulder—with solid circles representing the main peak and the squares representing the shoulder, and increasing power as lighter to darker shades. A third-order (second-order) power law is shown as a gray dashed (dotted) line. (c) TH diffraction contrast Δ_*I*_ plotted as dots connected with solid lines, as a function of pump pulse tuning and power, with light to dark colors representing increasing pump power (average pump power 0.7–5.2 mW in linear steps). The gray dashed line represents the TH diffraction contrast of the vertical orders, which fluctuates around 0, with a small deviation at lower input power that can be attributed to noise.

Given that the Fano resonant response at the fundamental frequency is by construction the result of two modes interfering, it is intuitive to expect that the third-harmonic response can also show multimode interference effects. In this view, tuning the pulse fundamental frequency will affect not just the overall TH generation efficiency but also the interference balance between contributions of the two modes to the nonlinear diffraction pattern. Indeed, tuning causes different complex superpositions of the two involved modes at the fundamental frequency to be excited, and so even in a perturbative picture (3ω curves are just the third power of induced polarizations at frequency ω), this should change nonlinear diffraction properties. Less obvious is how this could result in a pump–power dependence of the diffraction patterns and spectra that are different from a simple overall third power scaling. We note that a better understanding of this physics is of high relevance for nonlinear metasurfaces. There have been many reports on record-high spectrally integrated conversion efficiencies (the highest values for pump pulses line width-matched to quasi-BICs) and successful demonstrations of, e.g., nonlinear holograms. The notion of a nonlinear hologram is associated with the notion that nonlinear diffraction patterns are *stable* against pumping conditions. Our work points to a marked spectral reshaping of nonlinear diffraction patterns in dependence on pump pulse tuning and pulse power. This could, at the same time, be limiting for applications (lack of control over nonlinear output) or be enabling as a means to nontrivially control nonlinear response.

We developed a semianalytical model for TH responses in an effort to identify the minimum ingredients required to generate observations such as ours. The starting point is a coupled oscillator model for the frequency response of the system at a fundamental frequency. We set up a two-oscillator model with input–output ports, such that a low *Q* mode is directly driven by a Gaussian input pulse, while the high *Q* mode that generates the Fano resonance in transmission is only indirectly driven through coupling of the two oscillators. The low *Q* mode we identify as the ‘bright’ ED resonance, while the high *Q* mode represents the 'dark’ MD mode. A detailed description of the nonlinear coupled oscillator model, including all relevant equations, is provided in the SI. While the model is easily solved in the spectral domain and can be accurately fitted to the experiments by adjusting the parameters (*Q*-factors, resonance frequencies, coupling strengths), for nonlinear calculations, we proceed with the time-resolved modal excitation coefficients *a*_1_(*t*) for the ED and *a*_2_(*t*) for the MD mode of the form *a*_1,2_(*t*) = *ã*_1,2_(ω)*F*(ω)*e* ^*i*ω*t*^, where *ã*_1,2_ are the modal frequency-domain response functions and *F*(ω) represents the infrared laser pulse spectrum. The top section of Figure 5a presents a representative time trace of an off-resonant (1334 nm) driving pulse, along with the corresponding temporal ringdown of *a*_1_ and *a*_2_. At this wavelength, it is evident that the bright mode *a*_1_ is directly excited by the input pulse, whereas dark mode *a*_2_ is only weakly excited through coupling with *a*_1_. Since the pulse bandwidths in our experiments are wider than the Fano resonance, both the bright and dark modes are excited. The dark mode, characterized by a high *Q*-factor, exhibits a long ring-down time, though its limited spectral overlap with the pump pulse reduces the excitation amplitude. Conversely, the broad ED resonance produces a ringdown signature only slightly longer than the incident pulse duration. The top section of Figure 5b shows time traces for a resonant pump (1414 nm) and the corresponding temporal ringdown of *a*_1_ and *a*_2_. In this case, for a few optical cycles, nearly all of the energy initially in *a*_1_ transfers to *a*_2_, leading to a strong excitation of *a*_2_, which subsequently exhibits a large excitation amplitude and prolonged ringdown.

**Figure 5:**
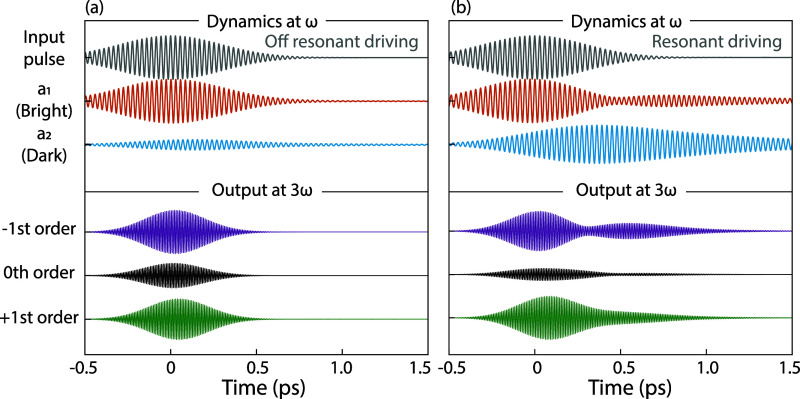
Coupled oscillator model dynamics: (a) In the top panel, the time evolution of the input pulse at off-resonant driving (1334 nm central wavelength) in gray and the ring-down of the two oscillators *a*_1_ (orange) representing the bright ED and *a*_2_ (blue) representing the dark MD. In the bottom panel, the time evolution of the conversion efficiency η at the third harmonic for the −1st (purple), 0th (black), and +1st (green) diffraction orders. (b) Temporal evolution for resonant driving with an input pulse centered at 1388 nm, the dynamics of *a*_1_ and *a*_2_ and at the bottom the conversion efficiency of the third-harmonic diffraction orders, indicated by the same colors as in panel (a).

To set up a simple description for the TH intensity in each nonlinear diffraction order, we evaluate a perturbative model, in which we assume that THG generation in the time domain is produced by a current distribution that is proportional to the third power of the local and instantaneous current distribution that is induced at the fundamental. In the modal Ansatz that is the coupled oscillator model, the temporal and spatial dependencies are by construction separated, meaning that the currents at the fundamental frequency are written as a product **j**_1,2_(*t*, *r*) = *a*_1,2_(*t*)*J*_1,2_(**r**), where the *J*_1,2_(**r**) are essentially the eigenmode spatial profiles. To obtain THG, we expand the superposition (**j**_1_(*t*, *r*) + (**j**_2_(*t*, *r*))^3^, as usual, keeping only the terms that lead to third-harmonic contributions (i.e., *a*_2_^3^, *a*_2_^2^ *a*_1_, *a*_2_ *a*_1_^2^, and *a*_1_^3^, but not contributions with a subset of the factors complex conjugated, which correspond to four-wave mixing). According to this logic, the intensity spectrum of any of the diffraction orders *m* = −1, 0, 1, expressed as TH conversion efficiency η, is of the form

$$\eta_{m}(\tilde{\omega })\propto |\int [A_{m}a_{2}(t{)}^{3}+B_{m}a_{2}(t{)}^{2}a_{1}(t)+C_{m}a_{2}(t)a_{1}(t{)}^{2}+D_{m}a_{1}(t{)}^{3}]e^{-i\tilde{\omega }t}dt{|}^{2}$$ 1

where ∫·e^–*i*ω̃*t*^d*t* arises to transform back to the spectral domain and the coefficients *A*_*m*_, ···*D*_*m*_ derive from the spatial distribution of currents Fourier transformed over the unit cell to obtain far-field diffraction efficiencies.

While a precise determination of the coefficients *A*_*m*_, ···*D*_*m*_ requires full-wave simulations, we argue that simple arguments inherent to the quasi-BIC nature of our structure already allow us to draw important conclusions. First, we expect the term proportional to *a*_2_^3^ to dominate for Fano-resonant metasurfaces: conversion efficiencies are boosted typically 10–100 times by the Fano resonance as compared to having just the broad ED mode (*a*_1_ contribution).^10,27^ This ratio depends on how well the incident pulse is line-width-matched to the Fano resonance at hand, with the highest values reported for picosecond pulses, while the lower value of 10 is pertinent to our experiment. This enhancement factor suggests a ratio |*a*_2_|/|*a*_1_| of order 10, and therefore, we take *D*_*m*_ small. Furthermore, we argue that the mixing terms containing both *a*_2_ and *a*_1_ cannot be ignored: if strictly only the dark high *Q* resonance would contribute to nonlinear diffraction, the relative intensity of diffraction orders would remain independent of the spectral content of the driving pulse. Instead, our key observation is that diffraction asymmetries depend on pulse tuning, implying that interference occurs from the different nonlinear contributions in eq 1.

Next, we examine spatial symmetries inherited from the original infrared modes by the nonlinear current distributions and thereby the diffraction intensity of opposing diffraction orders. The bright mode (*a*_1_ term) corresponds to an in-plane ED, which, in linear optics, gives rise to an even symmetry in the far-field radiated field. Instead, the dark mode corresponds to an out-of-plane MD term, which, in the far-field, gives rise to an odd-symmetry electric field. These symmetries carry over to the nonlinear terms, where terms that contain odd powers of *a*_2_ result in odd-symmetry diffracted far-fields, while terms with even powers of *a*_2_ result in even-symmetry contributions. Thus, each term in eq 1 taken individually would correspond to equal diffraction amplitudes at positive and negative diffraction angles; however, taking the terms together would show either constructive or destructive interference in opposing diffraction orders. Based on these principles, the TH conversion efficiency in the +1 and −1 diffraction orders, η_±1_, must be related as

$$\eta_{\pm 1}\propto |\int [\pm A_{1}a_{2}(t{)}^{3}+B_{1}a_{2}(t{)}^{2}a_{1}(t)\pm C_{1}a_{2}(t)a_{1}(t{)}^{2}+D_{1}a_{1}(t{)}^{3}]e^{-i\omega t}dt{|}^{2}$$ 2

whereas for the zeroth order, because of the same symmetry reasons, there would be no contribution of odd powers of *a*_2_, and the only terms left are *a*_1_^3^ and *a*_2_^2^ *a*_1_

$$\eta_{0}\propto |\int [B_{0}a_{2}(t{)}^{2}a_{1}(t)+D_{0}a_{1}(t{)}^{3}]e^{-i\omega t}dt{|}^{2}$$ 3

The bottom sections of Figure 5 show the resulting time traces for the ±1 and zeroth diffraction orders, highlighting the integrand in eq 2. The free parameter choices (*A*, *B*, *C,* and *D*) are discussed later, and a parameter study is presented in the SI. The ringdown traces (Figure 5) show beating patterns that are different in each diffraction order. This difference translates into different diffraction efficiencies for the two opposing diffraction orders.

While this perturbed coupled oscillator model brings out most of the salient physics, namely, the possibility of spectral/temporal shaping of THG in each order, and brings out the possible asymmetry in diffraction efficiency at opposing angles, it fundamentally cannot account for any nontrivial power dependence. By construction, all mode amplitudes in this model scale with the input field, and thereby, all terms have a common scaling with the input power. This leads to a model in which the overall conversion efficiency will follow a third-power law, but the way in which spectra reshape or diffraction efficiencies distribute asymmetrically is independent of power. To impart a power dependence on the model, we take inspiration from the measurement in Figure 4b, in which the conversion efficiency is observed to saturate at high intensities, and to do so at lower intensity for the narrow resonance as compared to the nonresonant contribution. This can be understood from the fact that if saturation arises from strong field effects, the large field enhancement at a narrow resonance will cause it to occur already at a lower incident power. We therefore insert a power-dependent saturation function *F*_*n*_(*P*), replacing each occurrence of *a*_*n*_(*t*) by *F*_*n*_(*P*)*a*_*n*_(*t*), with *P* being the input power. Inspired by the measurement in Figure 4b, which reveals a second-order power law dependence for the shoulder, we impose that

$${\left(F_{n}\right)}^{2}=\frac{F^{2}}{1+{\left(F^{2}/\left(F_{0}^{2}/Q_{n}\right)\right)}^{1/3}}$$ 4

where *F* is the power-dependent variable that follows $F=\sqrt{P}$ , *Q*_*n*_ is the quality factor *Q* for oscillator *n*, and *F*_0_^2^ is the saturation crossover power. Effectively, this power-dependent function tends to grow as *P* for low power or low *F*, and as *P* ^2/3^ for high power or high *F*, as to compute the TH output, the amplitudes enter as (|*a*_*n*_|^2^)^3^. The crossover power for each oscillator *n* is determined by *F*_0_^2^ corrected by *Q*_*n*_, meaning that the high-quality factor mode *a*_2_ is already entering the quadratic regime at a $\frac{Q_{2}}{Q_{1}}$ -times lower pump intensity than *a*_1_. The crossover value is defined as *F*_0_^2^ = 20 mW, which is analogous to the crossover powers of [Fig fig4]b, where the main peak grows cubically until 2 mW (and *Q*_1_ ∼ 10), whereas the shoulder shows deviating behavior at the lower end of the input power (*P* ∼ 0.1 mW, *Q*_2_ ∼ 300). With this empirical assumption, we attempt to replicate the data by fitting the free constants *A*, *B*, *C,* and *D*, while the oscillator parameters (including *F*_0_ and input power *P*) are taken from measured data.

Figure 6a presents the calculated TH spectra using this model, obtained by Fourier transforming the ring down traces for each order and summing the resulting spectral intensities over all diffraction orders (±1 and 0). The colors in the figure correspond to different pulse central wavelengths, similar to Figure 3b. The oscillator parameters for *a*_1_ and *a*_2_ were extracted by fitting to experimentally measured linear transmission spectra (as shown in Figure 2b), while the incident pulse parameters are obtained from Gaussian fits to the measured excitation pulse. All other model parameter values are given in Table S1 in the SI. Comparing the calculated TH spectra of Figure 6a with the measured TH spectra of Figure 3b, two key similarities stand out: (i) the presence of a shoulder near the Fano resonance and (ii) the highest TH conversion efficiency occurs when the pulse is tuned at the resonance. Calculated power-dependent spectra for two incident pulse tunings are shown in Figure 6b, describing the experiment in Figure 4a, for *F* ranging from $\sqrt{0.36}$ to $\sqrt{2.01}$ , matching the input powers in the experiment, for light to darker colors. The calculated spectra closely mirror the experimental observations, where the shoulder diminishes at higher input powers and the TH peak shifts away from the Fano resonance. The calculated TH contrast as a function of driving pulse tuning is plotted for six input powers with curves shaded from bright to dark, corresponding to increasing input powers (0.7–5.2 mW, $F=\sqrt{0.7}$ to $\sqrt{5.2}$ ) in Figure 6c. Although the power dependence is much less pronounced in the calculation, the experimental behavior is qualitatively well reproduced, allowing us to identify the minimal contributors to the observed effects: (1) Spectral reshaping occurs because THG arises not solely from the narrow resonance but also from its interference with THG from the broad resonance; (2) the different symmetries of the two modes lead to nonlinear interference, resulting in an asymmetric diffraction pattern; and (3) saturation of THG generation efficiency with increasing local field introduces a power dependence in both spectral reshaping and diffraction that reduces the cubic law to quadratic for higher input powers, where specifically the dark mode *a*_2_ crossover happens at $\frac{Q_{2}}{Q_{1}}$ -times lower pump intensity than for the bright mode *a*_1_. The model outlined above is based on simple principles, with the goal of demonstrating the minimum physical ingredients that capture the key mechanisms shaping TH spectra and diffraction across different input pulses, providing an initial understanding of the underlying physics. One might be tempted to view the model as a fitting description of the data, with the possibly complex-valued coefficients *A*, *B*, *C,* and *D* as free fit parameters. While we strongly caution against overinterpreting multiparameter fits to data of such simplified models, we argue that one can indeed adjust the parameters to obtain a consistent parametrization of our data set, from which one could perhaps extrapolate to other driving conditions or metasurface asymmetries. An exploration of calculated TH spectra and power-dependent contrasts, for different parameter choices *A*, *B*, *C,* and *D*, and pulse chirp and duration, is found in the SI.

**Figure 6:**
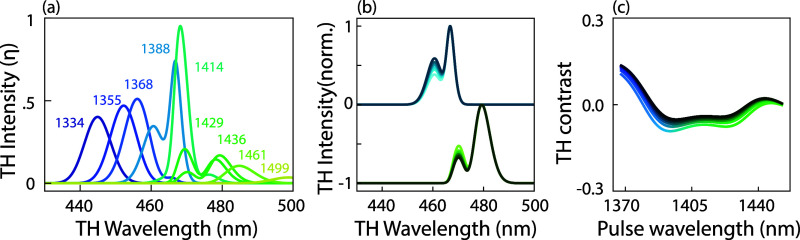
Modeled third-harmonic spectra and diffraction contrast as a function of input power. (a) Modeled TH spectra as a function of pulse tuning, with each color representing an excitation pulse centered at the wavelength indicated above the spectrum, closely resembling the experimental results from Figure 3b. The TH spectra are obtained from the Fourier transform of the combined ringdown in all diffraction orders. (b) Calculated power-dependent TH spectra for input pulses centered at 1388 nm (blue) and 1436 nm (green), where increasing power (lighter to darker shades) is modeled by increasing *F* in the power-dependent saturation function *F*_*n*_(*P*), where $F=\sqrt{P}$ , with *P* = 0.36, 0.55, 0.70, 0.88, 1.16, 1.47, and 2.01 mW. (c) Calculated TH diffraction contrast as a function of pulse central wavelength, plotted for six values of $F=\sqrt{P}$ , for *P* increasing linearly from 0.7 to 5.2 mW.

To further investigate the power dependence of third-harmonic (TH) diffraction patterns, we fabricated and analyzed a series of metasurfaces with varying asymmetry parameters α ranging from 0 to 0.4. We refer back to Figure 2 for the simulated and experimental linear responses of these metasurfaces as gauged by transmittance spectra. Figure 7a–c summarizes measurements of the TH diffraction contrast, quantified as the intensity ratio between the +1st and −1st diffraction orders, presented as a function of pulse tuning on the horizontal axis and asymmetry parameter on the vertical axis. Panels (a), (b), and (c) correspond to different average input powers: 1.4, 3.3, and 5.2 mW (0.37, 0.87, and 1.37 mJ/cm^2^), respectively. The color scale spans a TH contrast from −0.3 (blue) to 0.3 (red), indicating a maximum contrast of 30%. Generally, the TH contrast is negative, following the tuning of the Fano resonance with meta-atom asymmetry, and turns positive at wavelengths on either side of the resonance, being more positive for shorter (bluer) pump wavelengths. For small asymmetry values—where the Fano resonance has the highest *Q*-factor and the weakest spectral overlap with the input pulse—the contrast is predominantly positive. However, we must note that at α ≤ 0.1, the overall TH intensity is very low, leading to larger measurement uncertainties. A notable trend is that increasing the pump power shifts the overall contrast toward positive values, especially for longer (redder) wavelengths, while specific contrast depends strongly on pulse tuning. We evaluated the model employing the identical fitted constants (*A*, *B*, *C,* and *D*) as in Figure 5, while using oscillator parameters derived from fitting to the linear transmission data shown in Figure 2d to calculate power-dependent TH contrast across the same range of excitation pulse tunings as the measurement. The results of the model, presented in Figure 7d–f, demonstrate a clear transition of the TH contrast from positive to negative as the excitation pulse is tuned through the resonance. The inflection point of this transition follows the tuning of the Fano resonance with meta-atom asymmetry. Additionally, the increase in the power dependence results in an overall positive shift in the contrast around the Fano resonance, as well as on both sides of it. This qualitative behavior is consistent between the experiment and the model, albeit quantitatively less pronounced in the modeled data for variation with increased driving power. The reduced strength of the calculated power dependence may arise from an inaccurate estimation of the power crossover point used to determine *F*_0_ or from a modest choice of the power-dependent saturation of eq 4. Both the saturation function and its parameters serve as a first simple Ansatz in our minimum assumption model to bring out the main physics. A refined and microscopic model is required for an improved quantitative correspondence. Furthermore, although the spectral resolution of the experiment is quite limited compared to the calculations, the measurements still clearly capture the essential qualitative features. In particular, the measured data demonstrate the tunable diffraction contrast through the Fano resonance and its characteristic power dependence, which our model aims to explain. Both the experimental and modeled results reveal that for specific pulse wavelengths and powers, the TH contrast undergoes a complete reversal. This reversal happens either across two distinct powers at a given wavelength or across two wavelengths at a fixed power. These results highlight the tunable nature of the nonlinear diffraction pattern, which can be precisely controlled by adjusting the driving pulse properties. The observed behavior underlines the crucial role of the interference between the dark and bright modes in shaping the dynamic, wavelength-dependent diffraction contrast, further reinforcing the model’s ability to capture these complex dynamics.

**Figure 7:**
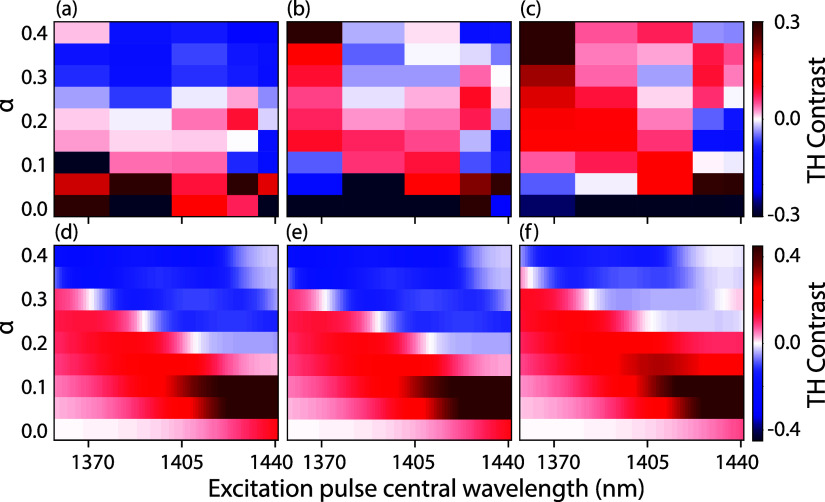
TH diffraction contrast across all fabricated asymmetry parameters as a function of five pulse tunings, with the color representing the TH contrast favoring the −1 (blue) and +1 (red) orders. The experimental results are shown in parts (a–c), where we vary the average pulse power as (a) 1.4 mW, (b) 3.3 mW, and (c) 5.2 mW (0.37, 0.87, 1.37 mJ/cm^2^ respectively). (d-f) Calculated TH contrast for oscillator parameters extracted from the experiment at similar pulse tuning range for *F* = $\sqrt{1.4}$ (d), $\sqrt{3.3}$ (e), and $\sqrt{5.2}$ (f), showing similar behavior as the experiment.

The crucial physics that explains the non-Gaussian shape of the TH spectra and the frequency-dependent TH contrast reversal for the diffraction orders is, according to our model, interference of the dark and bright mode contributions in the nonlinear far-field diffraction orders. An essential ingredient in this explanation is the phase relation between the bright mode *a*_1_ and the dark mode *a*_2_ mode, where the Fano resonance term *a*_2_ picks up a π phase shift when tuning through resonance. This transduces to a phase shift as a function of tuning for the nonlinear diffraction orders, which in turn contributes to the pump-wavelength dependence of the diffraction asymmetry. This rather indirect chain of reasoning has a signature that is directly accessible in the experiment. The phase shift is accessible in our experimental setup by performing wavelength-dispersed real-space imaging, wherein the 0th, +1st, and −1st orders are all recombined to produce interference. We project a slice of the real-space TH signal on the entrance slit of our grating spectrometer, with the asymmetry in the meta-atom aligned along the axis of the slit and the incoming light polarized perpendicular to it. Figure 8a presents dispersed real-space images for the same two pulses as in Figure 4a (1388 and 1436 nm central wavelength, top and bottom panels, respectively) at a pulse power of 0.7 mW. It is important to note that the asymmetry of the meta-atom, and thus, the slit and the imbalanced ±1st orders are oriented along the vertical *y* direction of the plots, while the *x*-axis corresponds to TH wavelength. Colors are plotted in a log scale to emphasize the low-intensity parts of the image. We indeed observe fringes in the spatial domain because of the interference of the 0th, +1st, and −1st orders. Summing intensity along the spatial coordinate should give the TH spectra: indeed, the two-component nature of the spectra (shoulders in Figure 4a) is evident, particularly in the bottom panel. The TH Fano resonance spans from 468 to 473 nm. The periodicity of the fringes along the spatial dimensions equals the metasurface pitch.

**Figure 8:**
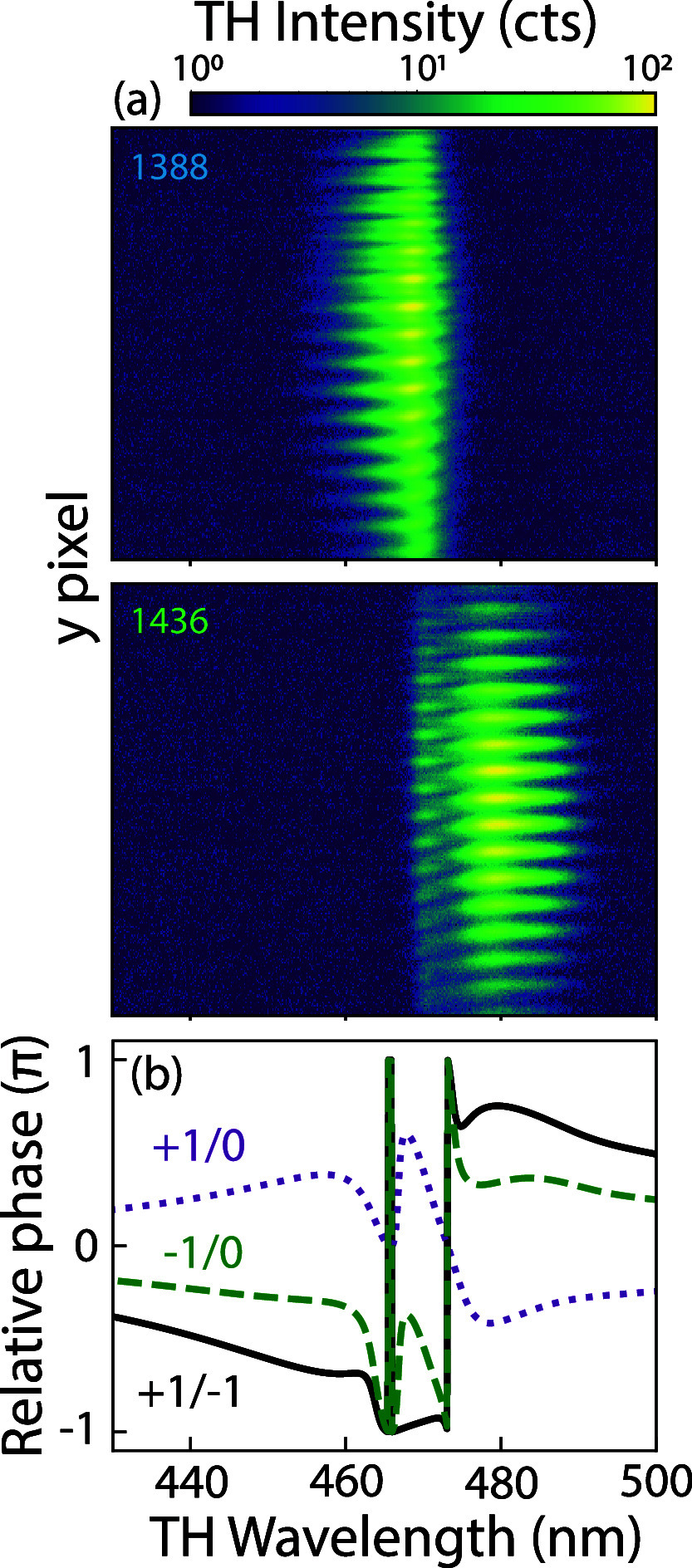
Observation of phase relation of the coupled oscillators: (a) Dispersed real-space images for a metasurface with asymmetry parameter α = 0.3 and pulse central wavelength = 1388 nm (1436 nm) on the top (bottom), showing clear phase jumps at the Fano resonance. TH intensity (counts) is plotted on a log scale to highlight the visualization at low intensity. (b) Calculated relative phase between the ±1st and 0th orders, showing a strong phase relation around the Fano resonance third-harmonic wavelengths, evidencing the influence of the phase relations of the diffracted orders to the TH spectra.

In these experimental images, the key observation that relates to the phase response of the Fano resonant contribution is a sudden spatial shift of the fringes by half a period, which is visible at/near the 468–473 nm wavelength range. This observation is direct evidence of the fact that the Fano resonant terms pick up a phase slip (*n*π for terms containing *a*_2_^*n*^). With the 0th-order light acting as a phase reference, this expresses as a shift in the fringes. Dispersed real-space calculations are presented in the SI, which reveal interference fringes similar to those in Figure 8a, including the phase slip at the TH of the Fano.

## Conclusions and Outlook

In summary, we presented observations of TH diffraction and spectra from Fano resonant metasurfaces and showed that TH diffraction imbalances and non-Gaussian TH spectra occur that are strongly dependent on pump pulse power and frequency tuning. These experimental observations were explored through a coupled oscillator model, which successfully replicated the phenomena. The observed TH diffraction imbalance is attributed to the constructive and destructive interference of nonlinear, mixed contributions from the two coupled oscillators in this model, and we argued how their radiation pattern properties result in asymmetries in TH conversion efficiency in opposing diffraction orders. This interpretation revolves around the idea that input pulse properties control the ring-down dynamics of the bright and dark modes responsible for the quasi-BIC Fano resonance characteristics, which, in turn, results, by nonlinear conversion, in multimodal interferences at the third-harmonic frequency. This view is supported by the fact that the signature π-phase slip in the resonant response of the dark mode is directly visible in the experimental data through real-space spectral measurements in which the diffraction orders interfere. Furthermore, we note that the observations point to the importance of saturation, with an increasing pump power of the TH efficiency. Indeed, power-dependent measurements revealed that the shoulder at the third harmonic of the Fano resonance reduces to a second-order power law, contrasting with the expected third-order behavior at pump powers much lower than the contribution of the bright mode. The calculated predictions align well with experimental observations, meaning that key features—(1) the asymmetric diffraction efficiencies, (2) the deviation from a purely third-order power law, and (3) non-Gaussian shaped TH spectra—can be explained in terms of a relatively simple coupled oscillator model.

An interesting question is how our findings translate to other quasi-BIC metasurfaces that support similar or different types of modes. By their very nature, quasi-BIC resonances exhibit an optical response that combines a broad continuum with a narrow resonance, creating a multimode platform that naturally enables spectral reshaping, nonlinear multimode interference, and intricate tuning dependencies. In addition, the metasurface period typically permits diffraction channels at the harmonic wavelength. While this is not a defining property of quasi-BIC metasurfaces, this condition is satisfied in most reported works on quasi-BICs. Another important characteristic is the difference in modal symmetry between the continuum and the narrow resonance, which drives diffraction asymmetries as a direct consequence of nonlinear multimode interference in the far-field. In this work, we focused on a system supporting bright and dark modes with symmetric and antisymmetric far-field emission, respectively. More generally, the rich landscape of quasi-BIC metasurfaces includes a wide variety of mode configurations involving both in-plane and out-of-plane field components. Many of these systems exhibit modal interference effects^4,26,28^ and are inherently subject to imbalanced diffraction physics, similar to our experimental observations and captured by our model. The symmetry-breaking route is currently the most used approach to achieve high-*Q* quasi-BIC metasurface resonances, and our findings generically apply to this class of metasurfaces. These symmetry-broken quasi-BICs can also host fundamentally different types of mode interactions and field distributions.^12,13,21,29^ In such cases, multimode interference in the nonlinear diffraction orders is still expected, though its expression will differ quantitatively.

An interesting avenue for future research is to incorporate the emission profiles of the modes at the harmonic wavelengths. In the present work, we focused on the emission profiles of the fundamental modes that, when raised to the harmonic order, interfere in the far-field diffracted orders. The fundamental modes consist of an in-plane ED and an out-of-plane MD that both yield a typical donut-like emission pattern, characterized by a node along the dipole axis. In the nonlinear diffracted orders, this leads to the even and odd symmetry arguments for the electric far-field. Obtaining the harmonic profiles of a single meta-atom would require full-wave simulations to extract the near-field current distribution. However, a full angular-dependent emission pattern is not required since intensity is guaranteed to only appear in the diffraction orders due to periodicity. Nonetheless, the inclusion of the emission profile of harmonic modes could provide a more complete picture of nonlinear diffraction, which provides more insight and furthermore expands the opportunities for nonlinear beam shaping and steering.

Our findings highlight the importance of driving pulse properties in the design of applications for nonlinear metasurfaces. This insight is highly relevant in the context of recent reports that focus on record-high spectrally integrated conversion efficiencies^30^ and nonlinear holograms that promise to produce robust diffraction patterns under varying pumping conditions.^31^ Our work reveals that nonlinear diffraction spectra can undergo significant reshaping, depending on the input pulse parameters. This spectral transformation presents both challenges and opportunities: on the one hand, it may introduce unpredictability in nonlinear output; on the other hand, it enables nontrivial control over nonlinear emission profiles. Crucial aspects that are not addressed in this work are the roles of pulse bandwidth, chirp, and the potential of dedicated pulse shaping. These aspects likely provide additional degrees of control over nonlinear diffraction, further emphasizing the necessity and potential of accurately tailoring driving pulse parameters for practical applications. These aspects hold promise for both fundamental studies, such as exploring advanced pulse shaping techniques for time-resolved studies and spectral–temporal control of nonlinear diffraction, and strategies for practical applications in tunable photonic devices, such as efficient optical routing^6^ or optical switching.^32^ Furthermore, insights from our coupled oscillator model suggest specific design strategies for creating efficient diffraction routers, such as *Q*-matching and aligning oscillator contributions. These methods could facilitate the development of advanced photonic devices, broadening the scope of metasurface applications by utilizing the deeper understanding of driving pulse properties to achieve tailored nonlinear responses.

## Supplementary Material



## Notes

Detailed nanofabrication procedure (I); rigorous description of the coupled oscillator model, including all relevant equations (II); experimental setup; and double Gaussian fits to TH spectra (IV) (PDF)
