# Supplementary material for: Influence of Driving Pulse Properties on Third-Harmonic Diffraction from Quasi-BIC Metasurfaces
Source: ACS Photonics. 2025 Nov 12;12(12):6620–30. doi: 10.1021/acsphotonics.5c01526 (PMC12715838; doi:10.1021/acsphotonics.5c01526)
Supplement: Supplementary file 1 [file ph5c01526_si_001.pdf]

Supporting Information: The Influence of Driving Pulse Properties on Third-Harmonic Diffraction from Quasi-BIC Metasurfaces

Falco Bijloo,[†,‡] Arie J. den Boef,[†] Peter M. Kraus,[†] and A. Femius Koenderink[*,‡]

†*Advanced Research Center for Nanolithography, Science Park 106, 1098 XG Amsterdam, The Netherlands*

‡*Department of Physics of Information in Matter and Center for Nanophotonics, NWO-I Institute AMOLF, Science Park 104, 1098 XG Amsterdam, The Netherlands*

¶*Department of Physics and Astronomy, and LaserLaB, Vrije Universiteit, 1081 HV Amsterdam, The Netherlands*

§*ASML Netherlands B.V., 5504 DR Veldhoven, The Netherlands*

E-mail: f.koenderink@amolf.nl

Number of pages: 18

Number of tables: 2

Number of figures: 5

I. Nanofabrication procedure

We employ a standard electron beamlithography (e-beam lithography) recipe to fabricate the nanostructures. Fused quartz substrates (12 × 12 mm, 500 μm thick, Siegert Wafer GmbH) were first cleaned by sonication in deionized water for 10 minutes. This was followed by immersion in a base piranha solution at 75°C for 15 minutes, then rinsed thoroughly with

water (2 dips of 15 seconds) and isopropanol (IPA). A 75 nm thick layer of polycrystalline silicon was deposited via e-beam evaporation (Polyteknik Flextura M508 E), using silicon pellets at an emission current of 90 mA, resulting in a deposition rate of 0.1 nm/s. To protect the silicon during later development steps and to improve adhesion to the resist, the samples were exposed to oxygen plasma for 2 minutes to grow a thin passivation layer. Next, a hydrogen silsesquioxane (HSQ) resist layer (Dow Corning XR-1541 E-Beam Resist) with an approximate thickness of 65 nm was spin-coated at 3000 rpm with an acceleration of 1000 rpm/s for 45 seconds, and baked at 180°C for 2 minutes. To suppress charging during e-beam exposure, a conductive Elektra layer (All Resist GmbH, Electra 92) was spin-coated on top at 2000 rpm, 1000 rpm/s, for 60 seconds and baked at 90°C for 2 minutes. Patterning was performed using a RAITH Voyager electron beam lithography system at 50 kV, with an average exposure dose of 1500 μC/cm^2. Development began with a 15-second dip in water to remove the Elektra layer, followed by 70 seconds in TMAH at 60°C, and rinsing in water and IPA for 15 seconds each. Finally, the HSQ mask pattern was transferred into the silicon layer using reactive ion etching (Oxford Instruments, Plasma Technologies Plasmalab 80 Plus) with a $CHF_3/SF_6/O_2$ gas mixture (15/10/3 sccm), at a forward power of 150 W and a chamber pressure of 7 mTorr. The etch rate under these conditions was approximately 45 nm/min. Residual resist was not removed, as it was found to have negligible effect on the optical properties or experimental outcomes.

II. Coupled oscillator model

Here, we report on the coupled oscillator model in fine detail.

Equations of motion

Suppose that you have a linear system of two coupled pendulums, leading to the following equations of motion

$$\begin{aligned} \ddot{a}_1 + \gamma_1 \dot{a}_1 + \omega_{0,1}^2 a_1 + \kappa a_2 &= \frac{F_1}{m} \\ \ddot{a}_2 + \gamma_2 \dot{a}_2 + \omega_{0,2}^2 a_2 - \kappa a_1 &= 0 \end{aligned} \tag{1}$$

where we will be assuming a_1 to be the broad oscillator (loss γ_1 of order $\omega_{0,1}/5$) of mass m that is directly driven by F_1, and where we assume a_2 to be the indirectly driven oscillator that couples to the driven oscillator, (with γ_2¡γ_1) which gives the Fano resonance. $\omega_{(0,n)}$ is the resonance frequency of oscillator n, and κ is the coupling between the two oscillators. For monochromatic driving oscillating of the form $e^{i\omega t}$, we derive to response functions that we call *polarizabilities*. These response functions are sometimes referred to as susceptibilities in literature; however, we shall avoid this terminology to disambiguate from nonlinear material susceptibilities. The polarizabilities are described as

$$\begin{pmatrix} a_1(t) \\ a_2(t) \end{pmatrix} = \begin{pmatrix} \alpha_1(\omega) \\ \alpha_2(\omega) \end{pmatrix} \frac{F_1}{m} e^{i\omega t} \tag{2}$$

with

$$\begin{pmatrix} \alpha_1(\omega) \\ \alpha_2(\omega) \end{pmatrix} = \begin{pmatrix} \omega_{0,1}^2 - \omega^2 + i\omega\gamma_1 & \kappa \\ -\kappa^* & \omega_{0,2}^2 - \omega^2 + i\omega\gamma_2 \end{pmatrix}^{-1} \begin{pmatrix} 1 \\ 0 \end{pmatrix} \tag{3}$$

These describe the frequency response in a single "modal excitation coefficient" α_n per oscillator n, where we introduced f that tunes leakage for in-output-port calculations. Depending on the parameter choice, this will give rise to Fano resonances in the spectral response, for instance, we visualize the oscillator excitations $\alpha_{1,2}$ by plotting both their absolute values in

Fig. S1, with α_1 increased 100-fold to enhance visibility. Furthermore, to calculate transmittance from $\alpha_{1,2}$, we start by defining $\mathbf{M}$ as the matrix on the RHS of equation 3, and adjust the driving of the first oscillator to include an input coupling rate that is consistent with the oscillator damping rate according to the requirements of coupled mode theory. One obtains a cavity response function of the form

$$\alpha(\omega) = 2i\omega\sqrt{\gamma_1} \cdot \left[\mathbf{M}^{-1}(\omega) \cdot \begin{pmatrix} 1 \\ 0 \end{pmatrix}\right] \tag{4}$$

which we can write out as

$$\alpha(\omega) = \frac{2i\omega\sqrt{\gamma_1}\left(i\gamma_2\omega - \omega^2 + \omega_2^2\right)}{\left(-\omega^2 + if\omega\gamma_1 + \omega_1^2\right)\left(i\gamma_2\omega - \omega^2 + \omega_2^2\right) - \kappa^2} \tag{5}$$

and the transmittance becomes

$$T(\omega) = \left|1 - \alpha(\omega)\sqrt{\gamma_1}\right|^2 \tag{6}$$

We fit this expression for the transmittance T to our experimental spectra to obtain the oscillator parameters (resonance frequencies, damping rates, coupling). Figure S1b) presents the fitted Fano lineshape to the experimental data, including spectra of the experimental and simulated driving pulses. Note that we could consider complex-valued couplings, which gives rise to the conjugate in equation 3. This complex coupling would result in electromagnetically induced absorption, as opposed to transparency. In our work we consider only real-valued coupling coefficients κ.

Gaussian pulse response

Let us move to discussing driving and response to pulsed excitation for the linear case, i.e. at the fundamental. We assume driving with a Gaussian pulse, such as in the experiment,

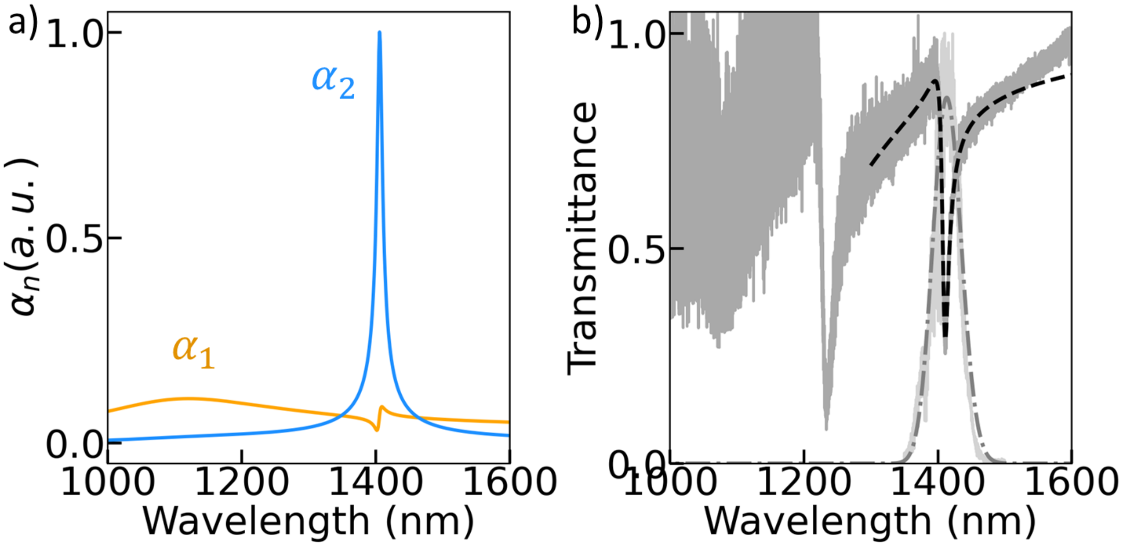

Figure S1: a) Modal excitation amplitudes α_1 (orange) and α_2 (blue) of the coupled oscillator model as a function of wavelength, showing the relative contribution of each mode to the overall response. b) Experimentally measured transmittance spectrum (grey) overlaid with the fitted Fano lineshape (black dashed), derived from the coupled oscillator susceptibility model, including experimental driving pulse on the background (lightgrey) and modeled driving pulse (darkgrey dash-dot). The broad oscillator α_1 is visible in the measured transmittance spectrum, even though with a low signal-to-noise ratio around 1100 nm, whereas the coupled narrow oscillator α_2 is visible near 1400 nm. A second Fano is visbile around 1250 nm, which could be attributed to a magnetic quadrupole or another higher order dark mode that couples to the same broad mode α_1

instead of monochromatic light. The driving will take the form

$$\frac{F_1(t)}{m} = e^{-t^2/2\Delta t^2}\; e^{i\omega_{0p}t}/\sqrt{\Delta t} \tag{7}$$

for a carrier frequency ω_{0p} and a pulse width Δt, the Fourier transform gives the spectrum, which for the field associated to the Gaussian pulse reads

$$F_1(t)/m = \int E(\omega)\, e^{i\omega t}\, d\omega \tag{8}$$

with

$$E(\omega) = \frac{1}{2\pi}\int e^{-t^2/2\Delta t^2}\; e^{i\omega_{0p}t - i\omega t - i\beta t^2}/\sqrt{\Delta t}\, dt \tag{9}$$

which is visualized in the top row of Fig. 5. Here, we introduced linear chirp β, which might be zero, but can give rise to a linear variation of the instantaneous frequency for nonzero values. The spectrum evaluates to $|E(\omega)| \propto e^{-(\omega-\omega_0)^2\Delta t^2/2}$, stating that the spectrum is Gaussian, centered around ω_0, and has a frequency width of $1/\Delta t$. If we apply this type of driving to the coupled oscillators, we obtain the following transient responses

$$\begin{pmatrix} a_1(t) \\ a_2(t) \end{pmatrix} = \int \begin{pmatrix} \alpha_1(\omega) \\ \alpha_2(\omega) \end{pmatrix} E(\omega)\, e^{i\omega t}\, d\omega \tag{10}$$

This gives us the temporal response of the oscillators, commonly referred to as the ringdown. From the ringdown, we can identify four obvious facts: 1) To feed amplitude into the Fano resonance, we need sufficient κ. 2) The ringdown of the high Q resonance α_2 is long, which means that many more cycles contribute to any harmonic generation compared to the ringdown of the broad mode α_1. 3) Unless enough energy is fed into the Fano resonance, the broad mode has a briefer flash but potentially higher modal intensity coefficient. Lastly, 4) matching pulse width and tuning affects the fraction of energy that feeds into the Fano. See Fig. 5 in the main text for ringdown calculations.

Third-harmonic generation

The coefficients $\alpha_{1,2}$(t) are the time-dependent modal excitation coefficients that describe the temporal response, while the real-space behavior is contained in electromagnetic mode profiles, which for modes 1 or 2 are associated with electric field distributions $\mathbf{E}_1(\mathbf{r})$ and $\mathbf{E}_2(\mathbf{r})$. This Ansatz means that the instantaneous field distribution induced in the sample at the fundamental frequency reads as a sum of factorized terms

$$\mathbf{E}(\mathbf{r}, t) = \mathbf{E}_1(\mathbf{r})\, a_1(t) + \mathbf{E}_2(\mathbf{r})\, a_2(t) \tag{11}$$

or in terms of the frequency domain polarizabilities

$$\mathbf{E}(\mathbf{r}, t) = \int \left[\mathbf{E}_1(\mathbf{r})\, \alpha_1(\omega) + \mathbf{E}_2(\mathbf{r})\, \alpha_2(\omega)\right] E(\omega)\, e^{i\omega t}\, d\omega \tag{12}$$

Third harmonic generation is understood to arise through a nonlinear material susceptibility, causing a local time-dependent nonlinear polarization to be induced everywhere where there is a nonzero nonlinear susceptibility $\chi^{(3)}$ (which we assume to be only in the Si meta-atoms)

$$\mathbf{P}_{\mathrm{TH}}(\mathbf{r}, t) = \chi^{(3)}(\mathbf{r})\, [\mathbf{E}(\mathbf{r}, t)]^3 \tag{13}$$

The induced nonlinear polarization can also be viewed as a nonlinear third-harmonic radiating current

$$\mathbf{j}_{\mathrm{TH}}(\mathbf{r}, t) = \frac{\partial \mathbf{P}_{\mathrm{TH}}}{\partial t} \tag{14}$$

It should be noted that we focus solely on $\chi^{(3)}$ effects and that the $[.]^3$ does not involve an absolute value of the field. Indeed, one should work with the physical field, i.e., just the the real part of the complex-valued electric field in the nonlinear polarization expression. This introduces the full richness of all possible combinations of $e^{\pm i\omega t}$ that arise when expanding the cubic term. This encompasses sum and difference frequency generation, effectively capturing the entire four-wave mixing landscape. Since we are specifically interested in frequencies near

the third harmonic, we narrow our focus and disregard the broader complexity of four-wave mixing, retaining only the +++ and — exponent terms that contribute directly to third-harmonic generation. This leads to a nonlinear current distribution of the form

$$\begin{aligned}\mathbf{j}_{\mathrm{TH}}(\mathbf{r},t) \propto \iiint \; & [\mathbf{F}_1(\mathbf{r})\alpha_1(\omega_1) + \mathbf{F}_2(\mathbf{r})\alpha_2(\omega_1)] \cdot [\mathbf{F}_1(\mathbf{r})\alpha_1(\omega_2) + \mathbf{F}_2(\mathbf{r})\alpha_2(\omega_2)] \cdot \\ & [\mathbf{F}_1(\mathbf{r})\alpha_1(\omega_3) + \mathbf{F}_2(\mathbf{r})\alpha_2(\omega_3)] \cdot \\ & E(\omega_1)E(\omega_2)E(\omega_3)\, e^{i(\omega_1+\omega_2+\omega_3)t}\, d\omega_1\, d\omega_2\, d\omega_3\end{aligned} \tag{15}$$

where the spatial dependence of χ is absorbed into the mode profiles $\mathbf{E}_1$ and $\mathbf{E}_2$ to get $\mathbf{F}_1$ and $\mathbf{F}_2$ To proceed, we must introduce further assumptions. If a_1 represents the broad mode and a_2 the narrow mode, then the nonlinear contribution from the broad mode $\mathbf{F}_1$ is expected to be significantly smaller than the contribution from $\mathbf{F}_2$ to the nonlinear current, due to the much weaker normalized field amplitude that is associated with the broad mode. This statement is quantitatively motivated by the observation that in Fano resonant metasurfaces harmonic generation efficiencies remains very low, unless the mode coupling constant and the excitation pulse width and tuning are selected to precisely match the quasi-BIC mode.Under this assumption, it is reasonable to expand the product and order the terms such that the $(\mathbf{F}_2)^3$ contributions lead, while also retaining terms with a single factor $\mathbf{F}_1$, i.e., terms proportional to $(\mathbf{F}_2)^2\,\mathbf{F}_1$, while discarding order contributions with multiple instances of $\mathbf{F}_1$. While in the description of THG Fano-resonant metasurfaces the discussion usually focuses on just the $(\mathbf{F}_2)^3$ term, we note that at least the next term is required to also describe the angle-dependent and pulse dependent effects that are the subject of our work. Note that because of the integration variables ω_n the expression can become quite inelegant to evaluate, and the ringdown traces and spectra of the third harmonic can have complicated shapes, arising from the frequency mixing of different frequency components. In our numerical calculations we perform a time-domain calculation

$$\mathbf{j}_{\mathrm{TH}}(\mathbf{r},t) \approx \left(\mathbf{F}_2(\mathbf{r})\right)^3 \left(a_2(t)\right)^3 + 3\left(\mathbf{F}_2(\mathbf{r})\right)^2 \mathbf{F}_1(\mathbf{r})\, a_2(t)^2 a_1(t) \tag{16}$$

which is a posteriori numerically Fourier transformed into frequency space

Diffraction order intensity asymmetry

Evaluating the THG diffraction efficiencies in Fano-resonant metasurfaces would require to numerically evaluate the far field radiated by each of the nonlinear current distributions in Eq. 16, which then need to be coherently summed to obtain the emitted intensity. In the limiting case where the contribution from the broad mode $\mathbf{F}_1$ vanishes, the radiation pattern arises solely from the narrow Fano resonance. If we associate the narrow mode to first order with an out-of-plane magnetic dipole mode, the resulting radiation pattern will be an angularly symmetric yet potentially spectrally structured third-harmonic response. This scenario provides a baseline explanation for experimental observations such as the reported spectral shape presented in Fig. 3, and supports the notion that spectral tuning of the excitation pulse to overlap with the Fano feature enhances overall THG efficiency. This response is modeled by considering the temporal overlap integral between the driving pulse and the resonant modes, as well as the dependence on the Fano Q-factor. Within this picture of just a single contributing mode, it is impossible to explain asymmetries in diffraction efficiency ratios, and also to explain any frequency structure therein as function of pulse tuning. This independence on pulse tuning is a direct result of the factorization in the temporal and spatial domain. Within this reasoning, the observed tuning-dependent asymmetry therefore must be a result of interference between multiple nonlinear contributions.

An important observation is that diffraction asymmetries and tuning dependencies do arise from the mixing of the first term with the second term in Eq. 16. As the laser is detuned, the relative phase and amplitude of the terms $\mathbf{F}_1$ and $\mathbf{F}_2$ evolve, due to the narrow spectral response of $\alpha_2(\omega)$ and the broader but nontrivial response of $\alpha_1(\omega)$. Moreover, the

two terms each individually present an angularly symmetric intensity distribution, but they do have different field symmetries. Because the intensity is calculated only after adding the contributing fields, this phase information is highly relevant, allowing for interference effects that manifest as asymmetry in the emitted TH diffraction pattern and spectra (Fig. 3), as well as the dispersed spatial jump (Fig. 8).

To understand the tuning dependence of the diffraction efficiency, one can begin by analyzing the symmetry properties of the radiation patterns associated with the nonlinear sources, which in turn arise from the linear currents $\mathbf{F}_1$ and $\mathbf{F}_2$. If the broad mode corresponds to an in-plane bright dipole, and the narrow mode to either an in-plane quadrupole or an out-of-plane magnetic dipole, their emitted field profiles have opposite symmetries (even vs. odd). Assuming the broad mode radiates a symmetric diffraction pattern with in-phase contributions to ± 1 orders, and the narrow mode contributes equal amplitude but with anti-phase components, diffraction asymmetry arises from their interference. With this reasoning, we conclude that for the plus and minus diffraction orders the efficiencies take the following form

$$\eta_{\pm 1} \propto \int \left| \int \left[\pm A a_2(t)^3 + B a_2(t)^2 a_1(t) \pm C a_1(t)^2 a_2(t) + D a_1(t)^3 \right] e^{-i\omega t}\, dt \right|^2 d\omega \tag{17}$$

and for the zeroth order

$$\eta_0 \propto \int \left| \int \left[B a_2(t)^2 a_1(t) + D a_1(t)^3 \right] e^{-i\omega t}\, dt \right|^2 d\omega \tag{18}$$

where we have retained all terms in expanding the cubic product. Here A, B, C and D are unknown parameters that in principle require full wave simulations of the metasurface resonances. These numbers may be complex valued. We treat them as parameters that are to be adjusted to phenomenologically describe the observed data.

Model parameters

The presented data in Figs. 5 through 8 in the main text, all use the following model parameters (Tab. S1):

Table S1: Unknown parameters used for model output data presented in Figs. 5-8 in the main text, and fitted pulse properties

**Unknown parameters**	**Pulse properties**
$A = -950 + 950i$	$\Delta t = 130$ fs
$B = 100 + 90i$	$\beta = -5 \cdot 10^{24}$ rad/s^2
$C = 50 - 50i$	
$D = 520 + 520i$	

In the main text we mention that the choices of parameters A, B, C and D strongly impact the modeled output. An optimization endeavor might be interesting to find best fits to the measured data, but is rather cumbersome and un-insightful. Therefore, we focused on comparing the key observations from our model to the measured data. To leave the reader with a minimum intuition to the impact of varying the model parameters, we provide an exploration of parameter dependencies in Fig. S2. Each panel shows TH spectra, power dependency and TH diffraction contrast calculations for varying a single parameter, while keeping the other parameters at their original value. For each parameter we provide results both for a a higher value and lower value compared to its original set value, as giving in the table below (Tab. S2).

Table S2: Exploration of the free model parameters with chosen high and low parameter values with their corresponding panels in Fig. S2, while other parameters are fixed as in Table S1

	**High**	**Panel**	**Low**	**Panel**
A	$-1550 + 1550i$	a	$-550 + 550i$	b
B	$150 + 140i$	c	$50 + 40i$	d
C	$80 - 80i$	e	$20 - 20i$	f
D	$750 + 750i$	g	$250 + 250i$	h
Δt	200 fs	i	60 fs	j
β	$-8 \cdot 10^{24}$ rad/s^2	k	$-3 \cdot 10^{24}$ rad/s^2	l

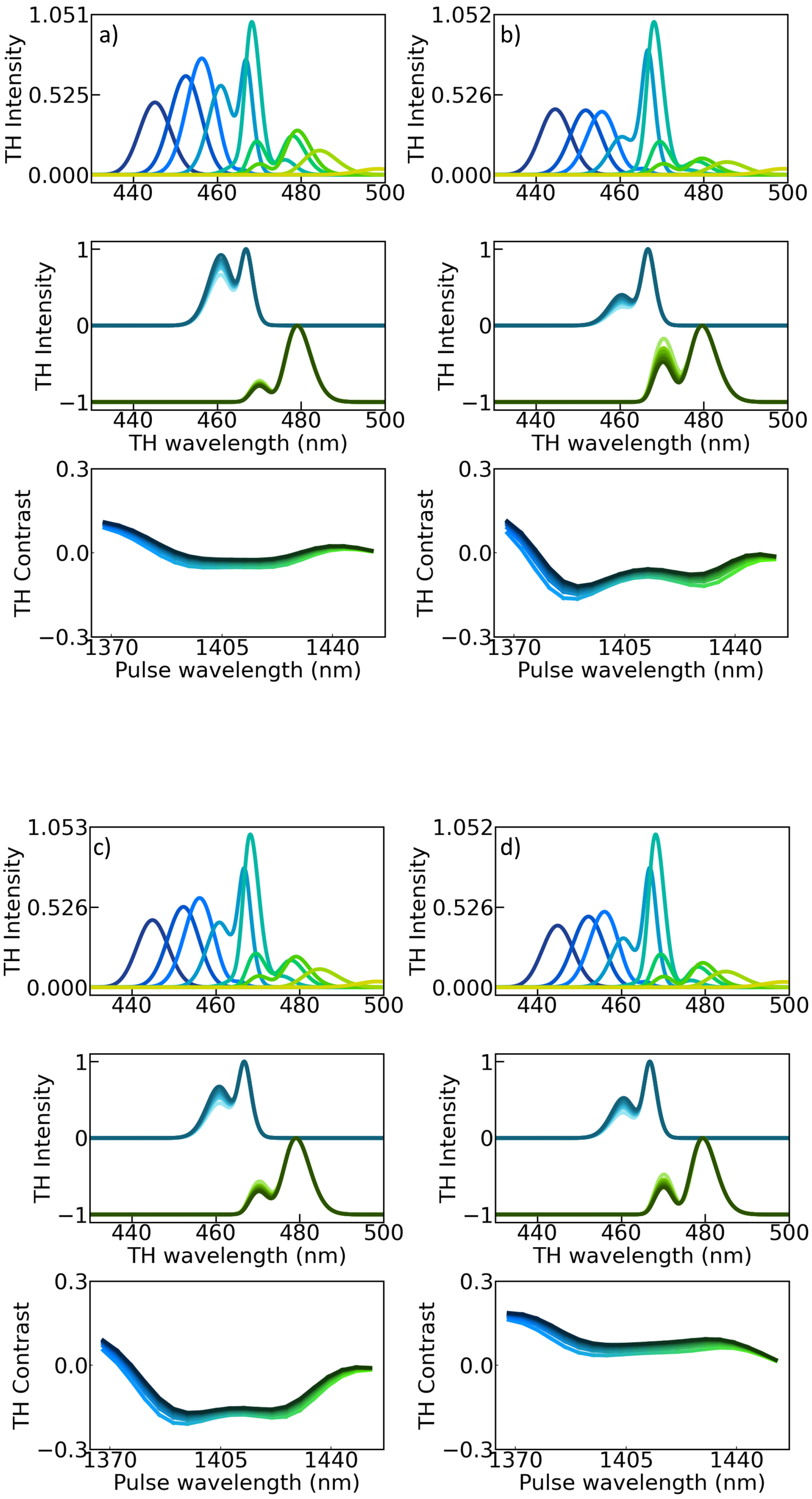
a)
b)
c)
d)
1.051
1.052
1.053
0.525
0.526
0.000
TH Intensity
TH wavelength (nm)
440
460
480
500
1
0
−1
TH Contrast
0.3
0.0
−0.3
1370
1405
1440
Pulse wavelength (nm)

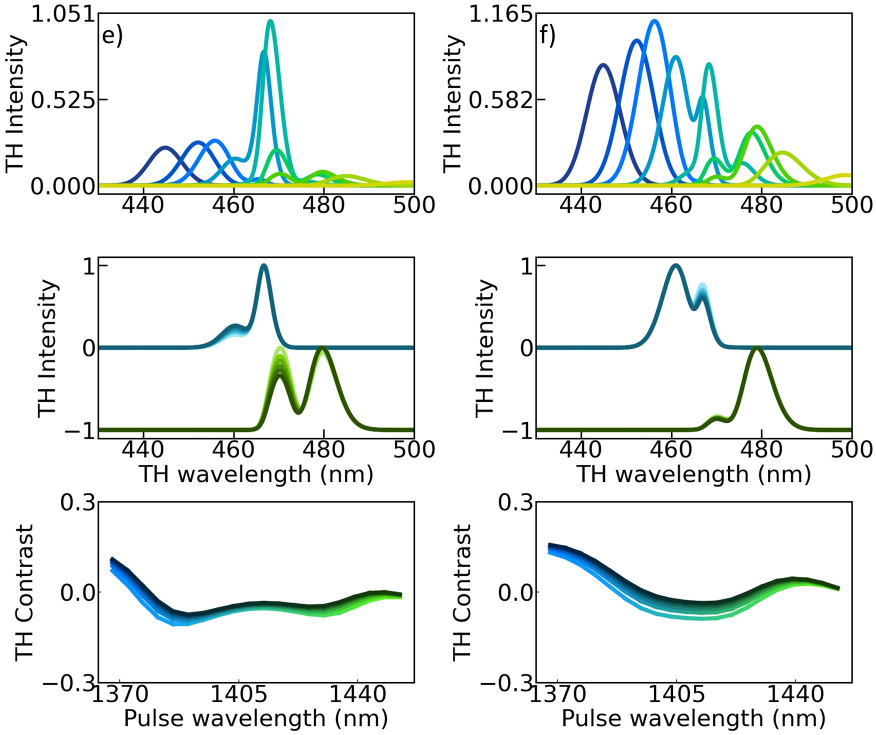

e)
f)
1.051
0.525
0.000
1.165
0.582
TH Intensity
440
460
480
500
1
0
−1
TH wavelength (nm)
0.3
0.0
−0.3
TH Contrast
1370
1405
1440
Pulse wavelength (nm)

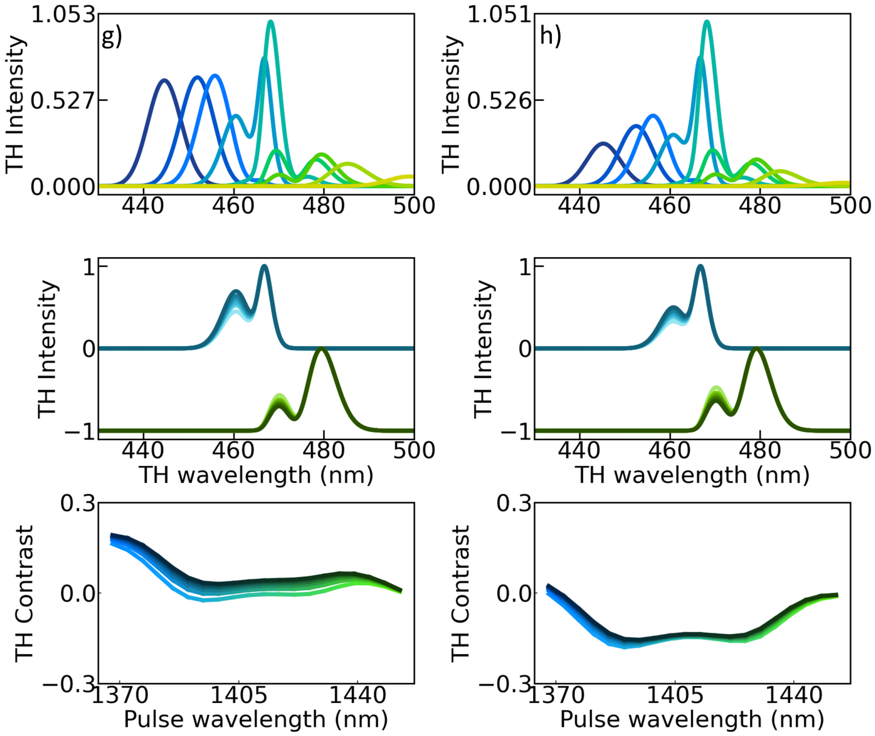

g)
h)
1.053
0.527
0.000
1.051
0.526
TH Intensity
440
460
480
500
1
0
−1
TH wavelength (nm)
0.3
0.0
−0.3
TH Contrast
1370
1405
1440
Pulse wavelength (nm)

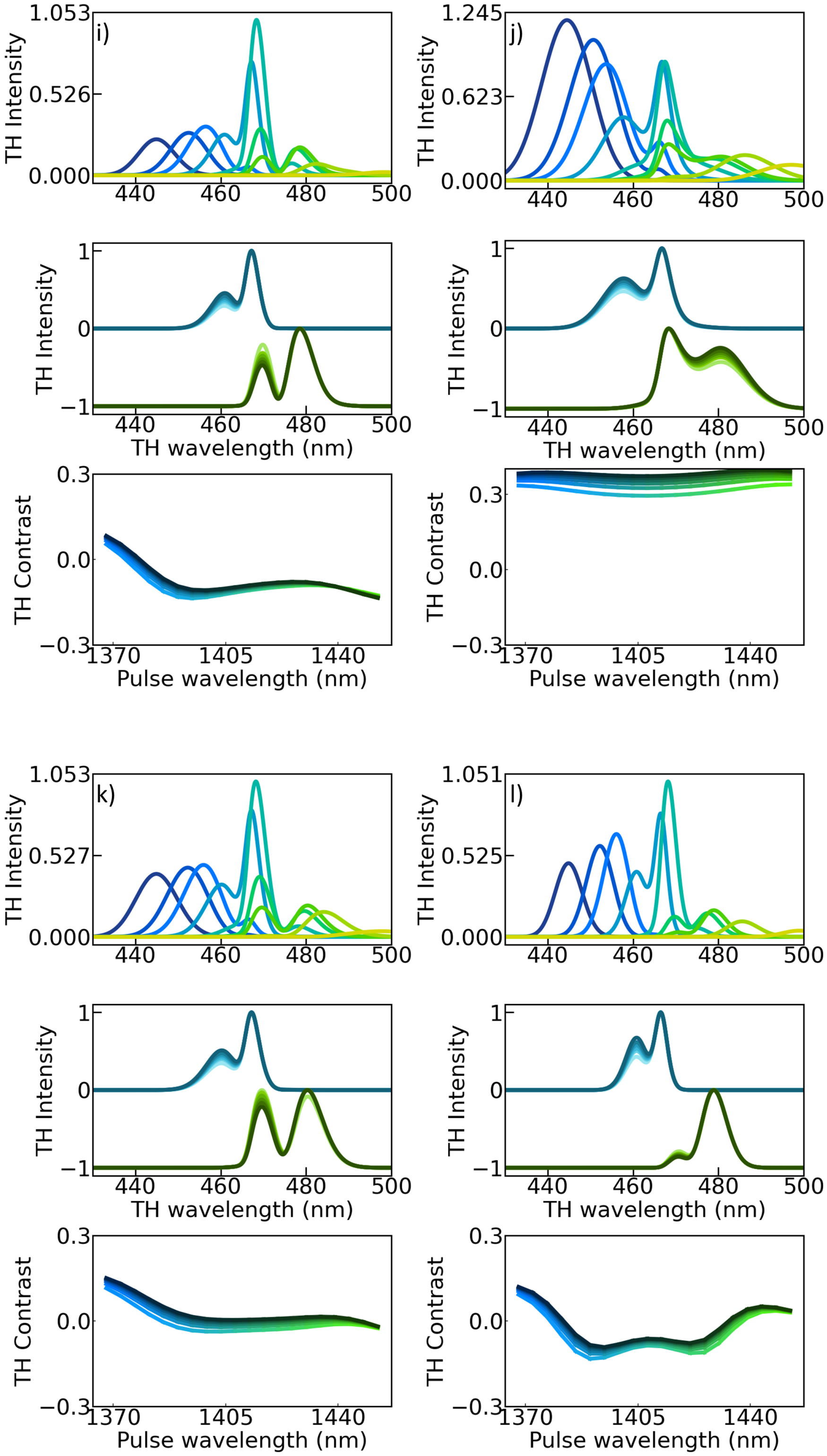

Figure S2: Exploration of varying the free parameters in the model. The results show TH spectra, power dependency and TH diffraction contrast, similar to the manuscript, for values given in table S2

Dispersed real-space calculation

The main text presents spectrally dispersed real space images in Fig. 8, highlighting phase jumps in the fringe pattern when traversing the wavelength range of the Fano resonances. Figure S3 shows a calculation, in which we form a real space image by summing three plane waves with similar amplitudes, that travel in the z-axis under an angle with the y-axis that is dependent on the metasurface grating pitch, exactly as measured in the Fourier space data. Interference between the three waves result from a phase-difference, that is calculated from Eqs. 17 and 18.

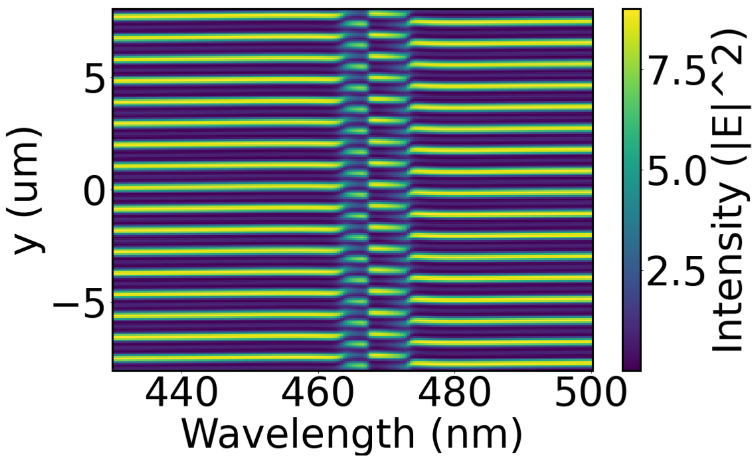

Figure S3: Dispersed real-space calculation. The three vertical diffraction orders 0, -1 and +1 interfere under an angle in the y-direction, that is given by the metasurface pitch. The intensity ($|E|^2$) is plotted in colorscale shown in the colorbar.

It is important to note the following differences between the measurement and simple model: 1) the measurement is performed with a spatial Gaussian intensity distribution and a Gaussian spectral distribution of the pump beam, whereas the simple model uses plane-waves with homogeneous amplitude over space and frequency, and 2) in this simple calculation, all three diffraction orders (-1,0,+1) carry a similar amplitude, instead of accounting for the actual diffraction efficiencies. However, the important feature in the dispersed real-space – the jumps in the fringe pattern at certain wavelengths – are independent of these assumptions.

III. Experimental setup

Our experimental setup (schematic setup in Fig. S4) produces 130 fs pulses at 1 MHz repetition rate that are generated in a LightConversion Orpheus OPA that is fed from a LightConversion Pharos 1030 nm laser. We use the idler to achieve pulses at the wavelength range of interest. A longpass filter of 1000 nm (Thorlabs FELH 1000) removes residual pump. We use a set of a halfwave plate (HWP, Thorlabs AHWP10M-1600) and a linear polarizer (LP, Thorlabs LPVIS050-MP2) to control incoming pulse fluence and polarization. An ND filter with OD = 1 (not shown) is placed after the linear polarizer, further reducing the pump power by one order of magnitude. A broad white light halogen lamp (AvaLight-HAL-S-Mini) can be coupled in to produce spectra at the fundamental wavelength. The beam is loosely focused by a f= 30C-coated lens (Thorlabs) and enters the sample from the backside. Emitted TH and transmitted IR pump, or white light, are collected in transmission side via a microscope objective (Nikon AC API plan, NA 0.9, 100×). A dichroic mirror (Edmund Optics 69-900) transmits the IR, that feeds into an optical spectrum analyzer (Thorlabs, OSA202C), and reflects the TH. The TH passes through a set of 2 IR filters (Thorlabs TF1), to further reduce pump intensity, after which it can be fed into a spectrometer (Ximea MC124MG-SY-UB) or directed to a visible camera (Teledyne Prime BSI Express). Via a 1:1 telescope that allows real- and Fourier space filtering, our so-called Fourier lens images the back focal plane of the objective. This allows to measure the angle resolved TH emission information. Without Fourier lens, the sample plane is imaged, creating real space TH images.

IV. Double gaussian fit to TH spectra

To track the main peak and the shoulder intensities in Fig. 4b), we fit a double Gaussian to the TH spectra. The double Gaussian is of the form

$$y(x) = H + A \exp\left(-\frac{(x-x_1)^2}{2\sigma_1^2}\right) + B \exp\left(-\frac{(x-x_2)^2}{2\sigma_2^2}\right) \tag{19}$$

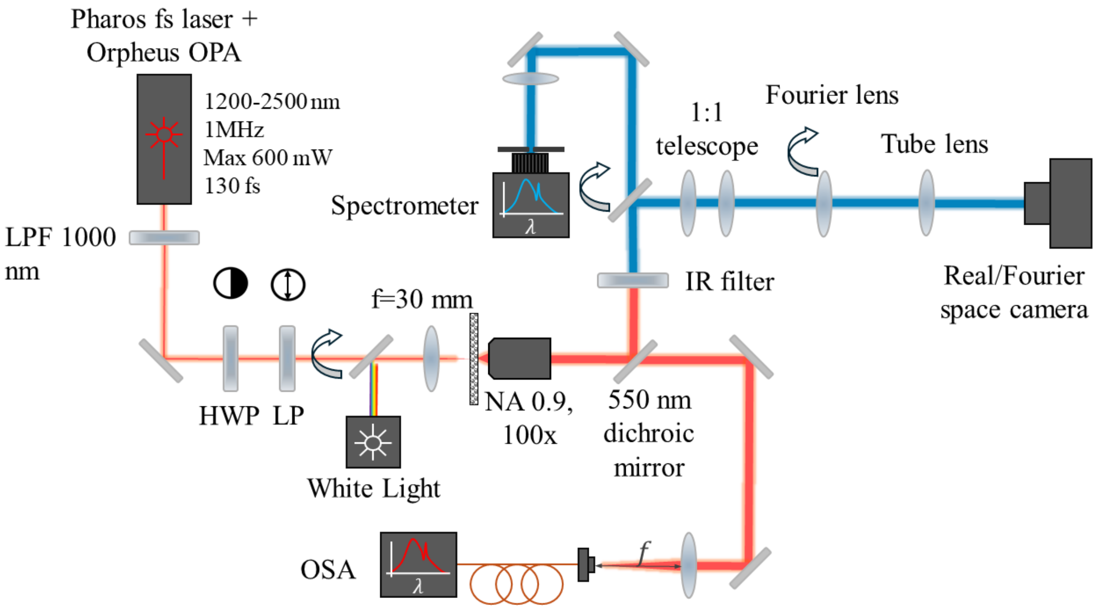

Figure S4: Experimental setup of our home-built nonlinear Fourier microscope.

with x the wavelength, y the TH intensity, H a constant baseline offset that is in our case close to 0, A the amplitude of the first Gaussian 'main' broad peak with x_1 the center wavelength that we keep close to the fundamental wavelength/3 and σ_1 the standard deviation that controls the peak's spread, and B, x_2 and σ_2 the amplitude, center wavelength and standard deviation of the second 'shoulder' narrow peak that is concentrated around the Fano wavelength/3. Figure S5 shows 6 example fits to measured spectra, that are also presented in the manuscript in Fig. 4. Figures S5a-c) show the double Gaussian fit to spectra acquired by exciting with a pump pulse centered at 1388 nm, at a) 0.3 mW, b) 0.6 mW and c) 1.1 mW in black solid, experimental in gray dots. The orange (blue) dashed curve depicts the main (shoulder) Gaussian associated to the output of mode a_1 (a_2), and the filled area beneath the curve indicates the integrated intensity that is plotted in Fig. 4b). Figures S5d-f) shows similar example fits, for a pulse that is centered at 1436 nm at d) 0.4 mW, e) 0.7 mW and f) 1.2 mW.

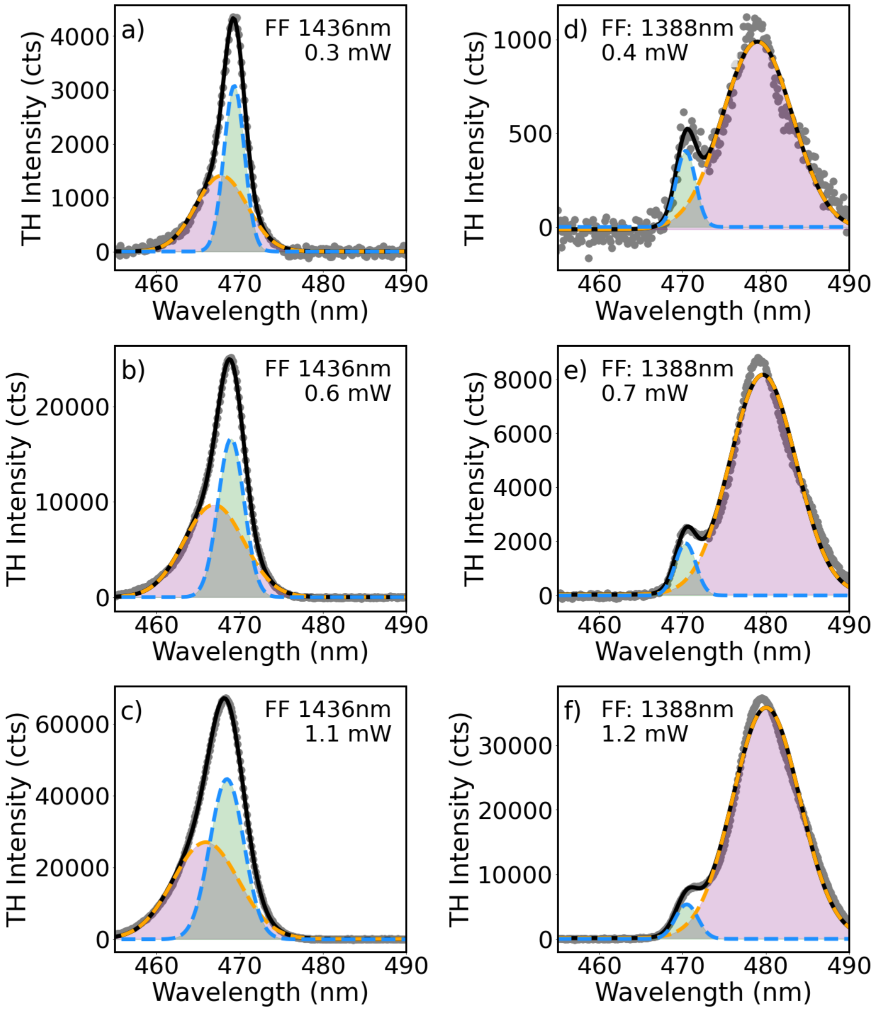

Figure S5: Double Gaussian fits to measured TH spectra. The measured spectra are plotted in solid grey dots, the double Gaussian fit in black solid, which is composed of two single Gaussians that are called the main peak in orange dashed and the shoulder in blue dashed. The integrated intensity of the peak is indicated by the marked area beneath the fits.
